# Long-term cost-effectiveness of matrix-associated chondrocyte implantation in the German health care system: a discrete event simulation

**DOI:** 10.1007/s00402-021-04318-9

**Published:** 2022-01-22

**Authors:** Tobias Vogelmann, Philip P. Roessler, Matthias Buhs, Sven Ostermeier, Justus Gille, Arnd Hoburg, York Zöllner, Sebastian Schwarz, Tino Schubert, Marco Grebe, Wolfgang Zinser

**Affiliations:** 1 LinkCare GmbH, Kyffhäuserstr. 64, 70469 Stuttgart, Germany; 2 Gelenkzentrum Mittelrhein GmbH, Mayen, Germany; 3 Norddeutsches Knorpelcentrum, Quickborn, Germany; 4 MVZ Gelenk-Klinik, Gundelfingen, Germany; 5 University Hospital Schleswig-Holstein, Campus Luebeck, Luebeck, Germany; 6 Med Center 360°, Berlin, Germany; 7 Hamburg University of Applied Sciences, Hamburg, Germany; 8 CO.DON AG, Leipzig, Germany; 9 OrthoExpert Fohnsdorf, Austria and GFO-Kliniken Niederrhein, Dinslaken, Germany

**Keywords:** Chondral defects, Knee replacement, Cost-effectiveness, Discrete event simulation, Autologous chondrocyte implantation

## Abstract

**Introduction:**

Cartilage defects in the knee can be caused by injury, various types of arthritis, or degeneration. As a long-term consequence of cartilage defects, osteoarthritis can develop over time, often leading to the need for a total knee replacement (TKR). The treatment alternatives of chondral defects include, among others, microfracture, and matrix-associated autologous chondrocyte implantation (M-ACI). The purpose of this study was to determine cost-effectiveness of M-ACI in Germany with available mid- and long-term outcome data, with special focus on the avoidance of TKR.

**Materials and methods:**

We developed a discrete-event simulation (DES) that follows up individuals with cartilage defects of the knee over their lifetimes. The DES was conducted with a status-quo scenario in which M-ACI is available and a comparison scenario with no M-ACI available. The model included 10,000 patients with articular cartilage defects. We assumed Weibull distributions for short- and long-term effects for implant failures. Model outcomes were costs, number of TKRs, and quality-adjusted life years (QALYs). All analyses were performed from the perspective of the German statutory health insurance.

**Results:**

The majority of patients was under 45 years old, with defect sizes between 2 and 7 cm^2^ (mean: 4.5 cm^2^); average modeled lifetime was 48 years. In the scenario without M-ACI, 26.4% of patients required a TKR over their lifetime. In the M-ACI scenario, this was the case in only 5.5% of cases. Thus, in the modeled cohort of 10,000 patients, 2700 TKRs, including revisions, could be avoided. Patients treated with M-ACI experienced improved quality of life (22.53 vs. 21.21 QALYs) at higher treatment-related costs (18,589 vs. 14,134 € /patient) compared to those treated without M-ACI, yielding an incremental cost‐effectiveness ratio (ICER) of 3376 € /QALY.

**Conclusion:**

M-ACI is projected to be a highly cost‐effective treatment for chondral defects of the knee in the German healthcare setting.

**Supplementary Information:**

The online version contains supplementary material available at 10.1007/s00402-021-04318-9.

## Introduction

Articular cartilage defects in the knee typically result from either degeneration or injuries. However, as articular cartilage is not supplied with blood, its ability to regenerate autonomously is limited and the defects are likely to progress to high-grade lesions if left untreated [1]. The most common symptoms of cartilage defects are pain, locking of the knee, and swelling. In the light of the latter, and under consideration of a high impact of articular cartilage defects on the quality of life [2] and productivity [3] of affected patients, the need for suitable treatment options is high. After a certain level of damage, conservative therapies are no longer sufficient, and surgery is unavoidable.

There are different treatment options available, in particular partial knee replacement (PKR), microfracture (MF), matrix-associated bone marrow stimulation (mBMS), autologous chondrocyte implantation (ACI), and matrix-induced autologous chondrocyte implantation (M-ACI). As a long-term consequence, osteoarthritis can develop over time and often leads to a need for total knee replacement (TKR) [4].

While the first generation of ACI consisted of chondrocytes being implanted under a periosteal flap and the second generation meant replacing this flap by a collagenous membrane, the third generation (M-ACI) is based on chondrocytes being seeded in a collagenous matrix. The latter is a fully autologous technique showing superior clinical results as compared to other methods, especially in larger lesions and regarding long-term outcomes [1, 5–8]. To the best of our knowledge, there are limited data available on long-term economic effects comparing PKR, microfracture, mBMS, and M-ACI. Yet, due to ethical considerations, aspects of practicability, and the time delay, the evaluation of long-term real-world data is not warranted. The aim of this study was, therefore, to evaluate the cost-effectiveness of M-ACI in Germany for treatment of articular cartilage defects in the knee using available data. We hypothesize that the longer the need for a TKR is deferred to the future, the higher the discounted net economic benefit.

## Methods

### Discrete event simulation model

This study used a discrete event simulation (DES) model to compare long-term economic consequences of M-ACI compared to other available treatments from a statutory health insurance (SHI) perspective.

DES is a form of computer-based modeling methodology which is characterized by the ability to simulate dynamic behaviors of complex systems and interactions [9]; it has a long history as a major tool for operational research and gains increasing application in the healthcare sector [10].

The goal of DES is to compare multiple treatment options to identify the most efficient ones and has compared with other modeling systems without interaction, the advantage to being able to accommodate complexities at the individual level instead of the overall cohort level [11]. DES can simulate the temporal sequence of events in cases where a direct measurement is not applicable due to time delays, practicability, or ethical considerations. Within this model, patients are considered as independent entities which are linked to adjunct information, such as age, sex, and morbidity characteristics [12].

### Model structure and study population

Table 1 and Figure S1 in the Supplemental material provide a schematic overview of the data sources and model architecture, respectively. Any individual enters the model with a symptomatic articular defect. Patient-level information about sex, age, and defect size of all patients treated with an M-ACI manufactured by CO.DON (chondrosphere® or Spherox®) between 2007 and 2021 were provided by CO.DON AG, Leipzig, Germany. Based on a random sample of these data (*N* = 10,000), sex, age, and defect size of the articular defect were assigned to each modeled individual. The model used a time horizon until death for each individual, during which time patients would die in line with the official German life tables [13]. The symptomatic individual received nonsurgical therapies, such as analgesia, nonsteroidal anti-inflammatory drugs, land-based exercise, and others, until a surgical option became available. Once the decision for surgical intervention was made, the individual would receive the surgery according to their defect size (see “Interventions” below for details). This primary intervention may either be successful, i.e., the individual won’t suffer any symptoms and the defect closes to 0 cm^2^, or unsuccessful. Unsuccessful interventions will keep the individual in a symptomatic health state until a successful intervention takes place. After a successful surgery of the defect, the implant will hold for a certain time and keep the individual symptomless until an event brings back a symptomatic defect. The process of surgery and implant failure will repeat for each individual until death. The model was run in two different healthcare scenarios: in the first scenario, matrix-induced autologous chondrocyte implantation is used in all patients as primary repair. This scenario is following the healthcare reality, since all patients entering the model were actually treated with M-ACI between 2007 and 2021. In a second, counter-factual scenario, M-ACI would not have been available to patients and patients would have been treated with other surgical or nonsurgical methods. All effectiveness and cost outcomes were computed by comparing the results from both scenarios which were run 10,000 times (once per patient) in both scenarios.

**Table 1:** Parameters and data sources

Parameters	Value	Source of information
Population inputs		
Population	Patient individual age, sex, defect size	CO.DON AG production data (data on file)
Mortality rate	Depending on age and sex	DESTATIS [13]
M-ACI		
Eligibility for defect sizes	> 2cm^2^	Clinical guideline [13]
Failure rate at 2 years	7%	[24, 25]
Long-term failure rate	–	Weibull estimation based on [24, 25] (see online appendix)
Costs	11,000 €	Administrative DRG data (I18A, I08G, ZE126) [26]
Microfracture		
Eligibility for defect sizes	≤ 2cm^2^	Clinical guideline [15]
Failure rate at 2 years	39%	[27]
Long-term failure rate	–	Weibull estimation based on [27] (see online appendix)
Costs	1500 €	Administrative DRG (I18B) [26] and outpatient renumeration data (EBM 31133/36133)
mBMS		
Eligibility for defect sizes	0–5 cm^2^	Clinical guideline [15]
Failure rate at 2 years	4%	[28, 29]
Long-term failure rate	–	Weibull estimation based on [28, 29] (see online appendix)
Costs	3500 €	Administrative DRG data (I18A) [26]
Partial knee replacement		
Eligibility for defect sizes	> 10cm^2^	Expert opinion
Failure rate at 2 years	4%	[20]
Long-term failure rate	–	Weibull estimation based on [20] (see online appendix)
Costs	6000 €	Administrative DRG data (I44E) [26]
Total knee replacement		
Eligible for defect sizes	> 10cm^2^	Expert opinion
Failure rate at 2 years	4%	[30]
Eligible age for TKR	55–80 years	Assumption
Long-term failure rate	–	Weibull estimation based on [30, 31] (see online appendix)
Costs	9000 €	Administrative DRG data (I44C) [26]
Mortality rate at surgery	Depending on age and sex, 0.0449–0.11%	Clinical studies and registry data [32, 33]

### Interventions

#### Microfracture

In microfracturing, a small bone defect is caused to trigger the repair mechanisms in a joint with cartilage damage. It is used primarily for deep cartilage damage that extends to the underlying bone. During the procedure, the cortical bone is perforated, or small holes are drilled. Blood leaking from the wound flushes stem cells to the surface, which adheres to the bone. Differentiation of these stem cells induces the formation of new cartilage tissue. Microfracture is usually performed during arthroscopy and is performed in two-thirds of cases on an outpatient basis and in one-third of the cases in an inpatient setting in Germany [14]. Symptomatic patients with defect sizes up to 2.0 cm^2^ are eligible for treatment with microfracture according to German treatment guidelines [15].

#### mBMS

Matrix-associated bone marrow stimulation (mBMS) is a modification of microfracture. After an arthroscopic assessment of the cartilage damage, an arthrotomy or arthroscopy is performed, and the shape and size are precisely measured. After that the subchondral bone is perforated and the matrix/membrane in the correct size is applied and fixed with sutures or fibrin glue. The post-treatment regimen depends on the localization of the cartilage damage. The average length of stay in the hospital is around two to three days, followed by rehabilitation. Patients need to go on crutches for about 6 weeks [16]. The SHI covers the costs of mBMS, which is usually performed in the inpatient setting.

#### M-ACI

Matrix-induced autologous chondrocyte implantation (M-ACI) is a further development of the ACI procedure and is also suitable for severe cartilage damage. In an initial arthroscopic procedure, the patient's own healthy cartilage is extracted. In special laboratories, cells are isolated from cartilage samples, placed in culture media (depending on the manufacturer) and propagated, and then implanted into matrices or transferred to three-dimensional chondrospheres. In a second surgical procedure, this matrix-ACI-product is transplanted back into the knee. In the following weeks and months, the cartilage cells continue to produce new cartilage tissue. Maturation of this tissue takes about 12 months. The post-treatment regimen; thus, depends on the localization of the treated cartilage damage. The average length of stay (LoS) in hospital is about two to three days, followed by rehabilitation. The German SHI pays for the costs based on two inpatient treatments as covered by DRG (Diagnosis Related Groups) system, plus an additional fee (‘Zusatzentgelt’). Symptomatic patients with defect sizes from 2.0 cm^2^ upwards are eligible for treatment with M-ACI according to German treatment guidelines.

#### Partial and total knee replacement

Knee replacement is one of the most common elective routine procedures in orthopaedic and trauma surgery in Germany, and worldwide, to treat patients with advanced osteoarthritis of the knee [17, 18]. The following main criteria should be present for the indication of TKR: (i) knee pain for at least 3–6 months, (ii) evidence of structural damage (osteoarthritis, osteonecrosis) proven by X-ray, (iii) unsuccessful conservative therapeutic measures for at least 3–6 months, and (iv) limitation of quality of life related to the knee joint disease. For the indication for TKR, there should be evidence of structural damage (osteoarthritis, osteonecrosis) [19]. The intervention is fully covered by the SHI system. The surgery itself lasts around 60–90 min, the average LoS in hospital is seven days, and after hospital discharge, patients undergo a three-week rehabilitation. Patients need to go on crutches for about four weeks to three months and will be unable to work (sick leave) for around three to six months, depending on the physical demands of their job. The average service life of a TKR is assumed to be around 25 years [20]. However, approximately 10–20% of patients are not or not completely satisfied with the outcome after the surgery [21, 22], which is taken into account by quality-of-life decrements in the model.

#### Modeling failure rates

The information on failure rates was obtained from follow-up studies in the international literature (see Table 1). We assumed separate Weibull distributions for short- and long-term causes for implant failures. The probability–density curves of the two Weibull distributions were then merged to a joint distribution. Weibull models were adjusted to fit the observed values by means of the Lifedata. MLE function of the SPREDA package in R 4.0.3, the free software [23]. Detailed information is provided in the supplementary material.

#### Health outcomes and costs

Health gains from the interventions were expressed as QALYs and were calculated using the following technique: Between two subsequent events, individuals would either stay in the health state of symptomatic defects or in the state without symptomatic defects; patients would gain QALYs for every year in the corresponding state until a new event occurred. QALY weights were taken from a previous assessment of an M-ACI technology by NICE [4], see Table 2.

**Table 2:** Health gains of the different interventions

Health state	QALY weights resp. gains per year
Before primary repair	0.65
Primary ACI, year 1	0.76
Primary ACI, years 2 +	0.82
Primary MF, year 1	0.76
Primary MF, years 2–4	0.82
Primary MF, year 5	0.65
Before 2nd repair	0.65
No 2nd repair	0.69
2nd repair ACI | 1st repair ACI, yr 1	0.76
2nd repair ACI | 1st repair ACI, yr 2 +	0.82
2nd repair MF | 1st repair ACI, yr 1	0.76
2nd repair MF | 1st repair ACI, yr 2–4	0.82
2nd repair MF | 1st repair ACI, yr 5	0.65
2nd repair MF | 1st repair MF, yr 1	0.76
2nd repair MF | 1st repair MF, yr 2–4	0.82
2nd repair MF | 1st repair MF, yr 5	0.65
No further repair	0.69
Before 1st KR	0.62
Successful 1st KR | TKR/PKR	0.78
Before 2nd KR	0.56
Successful 2nd KR	0.78
No further KR	0.69

*ACI* autologous chondrocyte implantation, *MF* microfracture, *KR* Knee replacement, *TKR* total knee replacement, *PKR* partial knee replacement

According to the German DRG catalogue and the billing catalogue for outpatient procedures (“Einheitlicher Bewertungsmaßstab”, EBM), the procedure costs for an M-ACI were 11,000 €, for an mBMS 3500 €, for a microfracture 500 €, for a TKR around 9000 €, and a PKR around 6000 € (Table 1). A revision of a TKR was assumed to be 15,000 €. Conservative therapy for a knee defect, e.g. physiotherapy and pain medication, was estimated to be 800 € per year.

#### Perspective and discount rates

The model was designed from the perspective of the German SHI system. Therefore, direct healthcare costs were considered, but no costs of working disability or early retirement were included in the model. Medical rehabilitation is partly funded by the pension insurance in Germany and was not considered. QALYs and costs were discounted a 2% discount rate in the base case scenario. As there is no official willingness to pay (WTP) threshold per QALY gained in Germany, we calculated a cost-effectiveness acceptability curve (CEAC), which shows the probability of M-ACI being a cost-effective option for different WTP thresholds.

#### Accounting for uncertainty

Uncertainty arises in the model in two ways: first, stochastic uncertainty and heterogeneity arising from structural or random variability in outcomes between patients. This uncertainty is typically accounted for in the context of Markov models with probabilistic sensitivity analyses (for example, Monte Carlo simulations). In the discrete event simulation used here, this uncertainty is already considered in the base case by 10,000 repetitions and the consideration of different individuals.

Secondly, parameter uncertainty arises. We have taken this into account by means of univariate sensitivity analyses, in which we have considered the influence of changes in assumptions on the outcomes. An one-way sensitivity analysis was performed and presented as tornado plot to assess the impact of a fixed change of ± 20% in input parameters on the ICER per QALY gained in the M-ACI scenario. As significant health gains and costs occur several decades after the initial surgery, especially costly TKR, and no official recommendation on discount rates in healthcare evaluations in the German system are present, we further assessed the impact of a discount rate of 4.5% for effects and costs. Also, since microfracture is commonly used in larger defect sizes [34], we also assessed a scenario where patients are eligible for microfracture for defect sizes as large as 5 cm^2^.

#### Model development and statistical analysis

The model was built in TreeAge Pro 2021, R1.2 Healthcare [35]. All input variables sourced from the literature were discussed among, and consented within, the author group, consisting of scientific and clinical experts in the treatment of chondral defects.

## Results

### Baseline characteristics

Upon entering the model, patients were 36 years old on average; approximately 60% were male; there was no significant age difference between men and women. Mean initial defect size was 4.2 cm^2^. 48.4% of patients had initial defect sizes smaller than 4 cm^2^ (see Table 3). Median life expectancy was 84 years, so that the DES covered a median time horizon of 48 years.

**Table 3:** Baseline characteristics in the discrete event simulation

	Total	Male	Female
*N*	10,000	6069 (60.7%)	3931(39.3%)
Age in years < 35 35– < 45 45– < 55 ≥ 55	36.1 ± 11.0 4531 (45.3%) 2955 (29.5%) 2245 (22.5%) 269 (2.7%)	35.9 ± 10.9 2833 (46.7%) 1823 (30.0%) 1258 (20.7%) 155 (2.6%)	36.6 ± 11.3 1698 (43.2%) 1132 (28.8%) 987 (25.1%) 114 (2.9%)
Defect size in cm^2^ < 2 2– < 4 4– < 6 6– < 8 8– < 10 ≥ 10	4.4 ± 2.6 1360 (13.6%) 3477 (34.8%) 2641 (26.4%) 1160 (11.6%) 982 (9.8%) 380 (3.8%)	4.5 ± 2.7 746 (12.3%) 2030 (33.4%) 1652 (27.2%) 745 (12.3%) 644 (10.6%) 252 (4.2%)	4.1 ± 2.6 614 (15.6%) 1447 (36.8%) 989 (25.2%) 415 (10.6%) 338 (8.6%) 128 (3.2%)

Values are mean ± standard deviation or number of patients (%). Percentage refers to column total, except percentage of *N*, which refers to line total

### Number of interventions and total knee replacements

Regarding the scenario with M-ACI use, a patient had his/her first intervention at the age of 36 years. 82% of the patients had a second intervention, the mean time to 2^nd^ intervention being 17 years. Of those, 33% renewed the M-ACI, 63% had other surgical procedures, and 4% underwent TKR. 60% of these patients underwent a third intervention, this mean time accounting for another 6.6 years. Until death, a total of 5.5% received a TKR at the average age of 65 years, 29 years after the first M-ACI. 13% of the patients underwent a revision surgery after a mean of 6 years after TKR. Until their death at the average age of 84 years, patients underwent 2.2 of the defined surgeries.

In the scenario without M-ACI, 35% of the patients underwent mBMS as primary intervention, while 24% underwent microfracture and 39% received other procedures, such as PKR. As in the scenario with M-ACI, patients had their first intervention at the age of 36 years and an average defect size of 4.2 cm^2^. 86% of the patients underwent a second intervention after on average 12 years. Of those, 39% underwent mBMS, 24% each received a microfracture or other surgical procedures, and 13% had a TKR. 66% of these patients underwent a third intervention after another 6.4 years. Until death, a total of 26% received a TKR at the average age of 62 years, 26 years after the initial intervention, and had, on average, three previous surgeries. 17% of patients who received a TKR underwent a revision surgery after a mean of 6 years after TKR. Until their death at the average age of 84 years, patients underwent 3.2 of the defined surgeries.

**Fig. 1:**
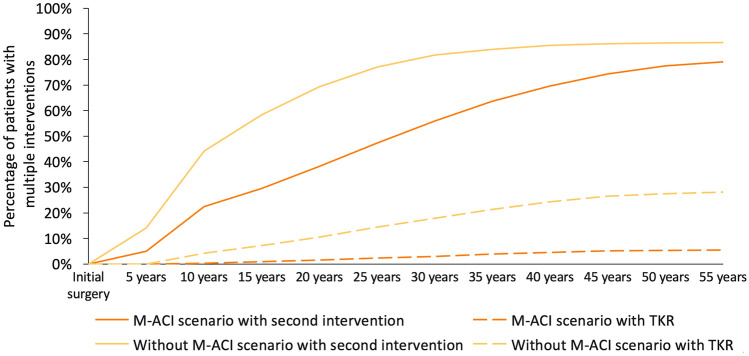
Percentage of patients with multiple interventions/TKR with M-ACI and without M-ACI

Observing the key figure of second surgeries, the percentage of patients with a second intervention increases over the years in both scenarios. At any time, the percentage of patients with a second intervention is significantly higher in the scenario without M-ACI; e. g. 25 years after the initial intervention, 47% of the patients with M-ACI underwent a second surgery compared to 77% of the patients without M-ACI. Regarding the prevention of TKR, it can be observed that more patients in the non-M-ACI scenario received a TKR during the observation period. Compared to the non-M-ACI patients, 21% of TKRs in the M-ACI scenario were prevented (see Fig. 1).

Regarding the number of surgeries before a TKR, it can be observed that more patients in the scenario without M-ACI have a TKR (5.5% in the scenario with M-ACI vs. 26% without), corresponding to a difference of 21%. When compared to the scenario with M-ACI, patients without M-ACI are, on average, one year younger when receiving their TKR.

### Quality of life, costs, and cost-effectiveness

**Fig. 2:**
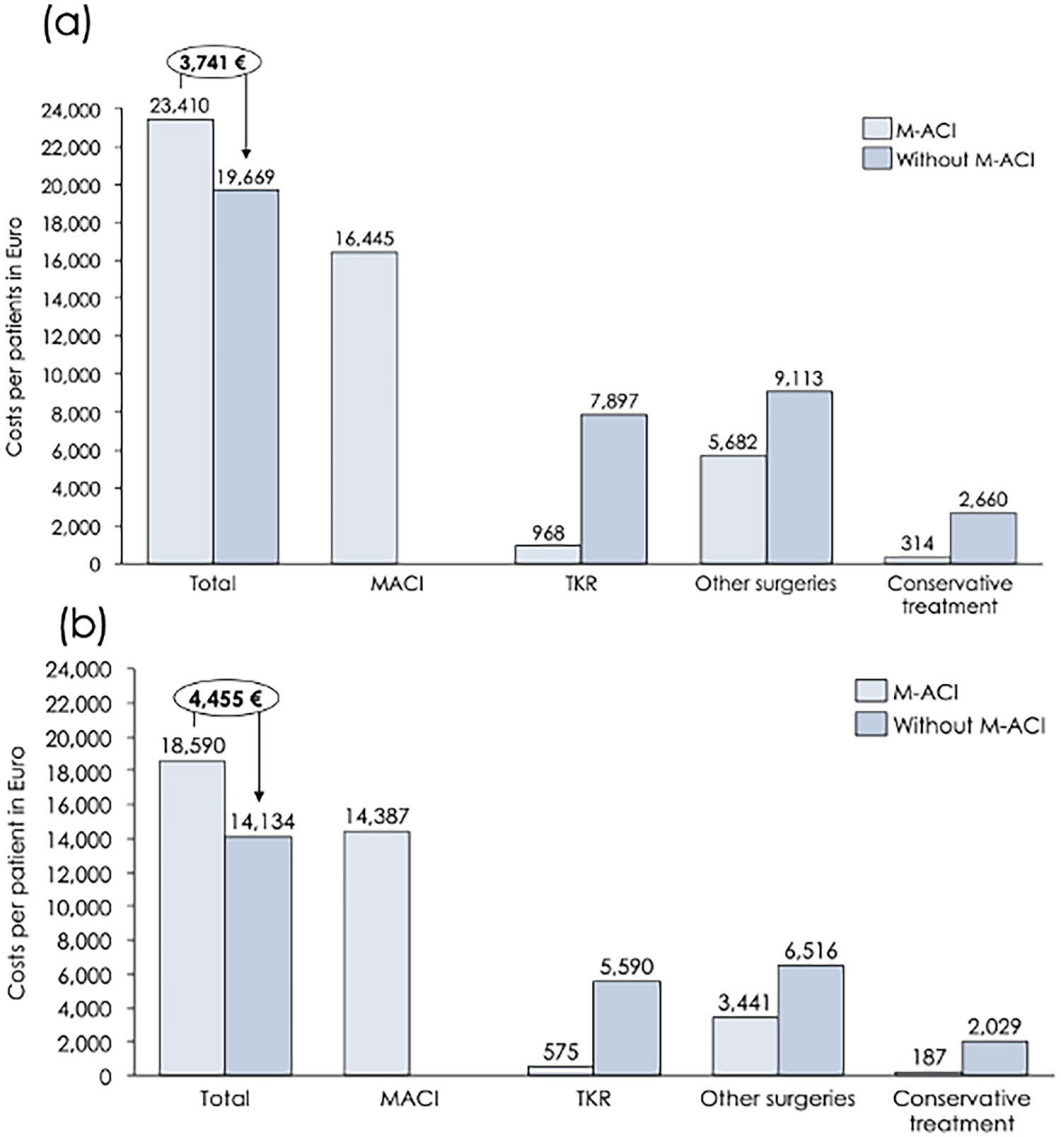
**a** undiscounted and **b** discounted total costs by treatment scenario; isolated M-ACI-only and other intervention costs; M-ACI vs. non-M-ACI net cost difference encircled

Total undiscounted costs in the scenario with M-ACI were 23,410 € and 19,669 € in the M-ACI and non-M-ACI scenario, respectively. The cost difference of 3741 € was mainly driven by the costs for M-ACI (16,445 €) in the M-ACI scenario which were partly offset by savings of 6929 € for avoided TKR. In discounted cost terms, costs were 18,590 € in the M-ACI scenario and 14,134 € in the scenario without M-ACI, yielding a difference of 4455 € (see Fig. 2).

Costs of the two scenarios converged notably during the follow-up period (see Supplemental Material Figure S2).

**Fig. 3:**
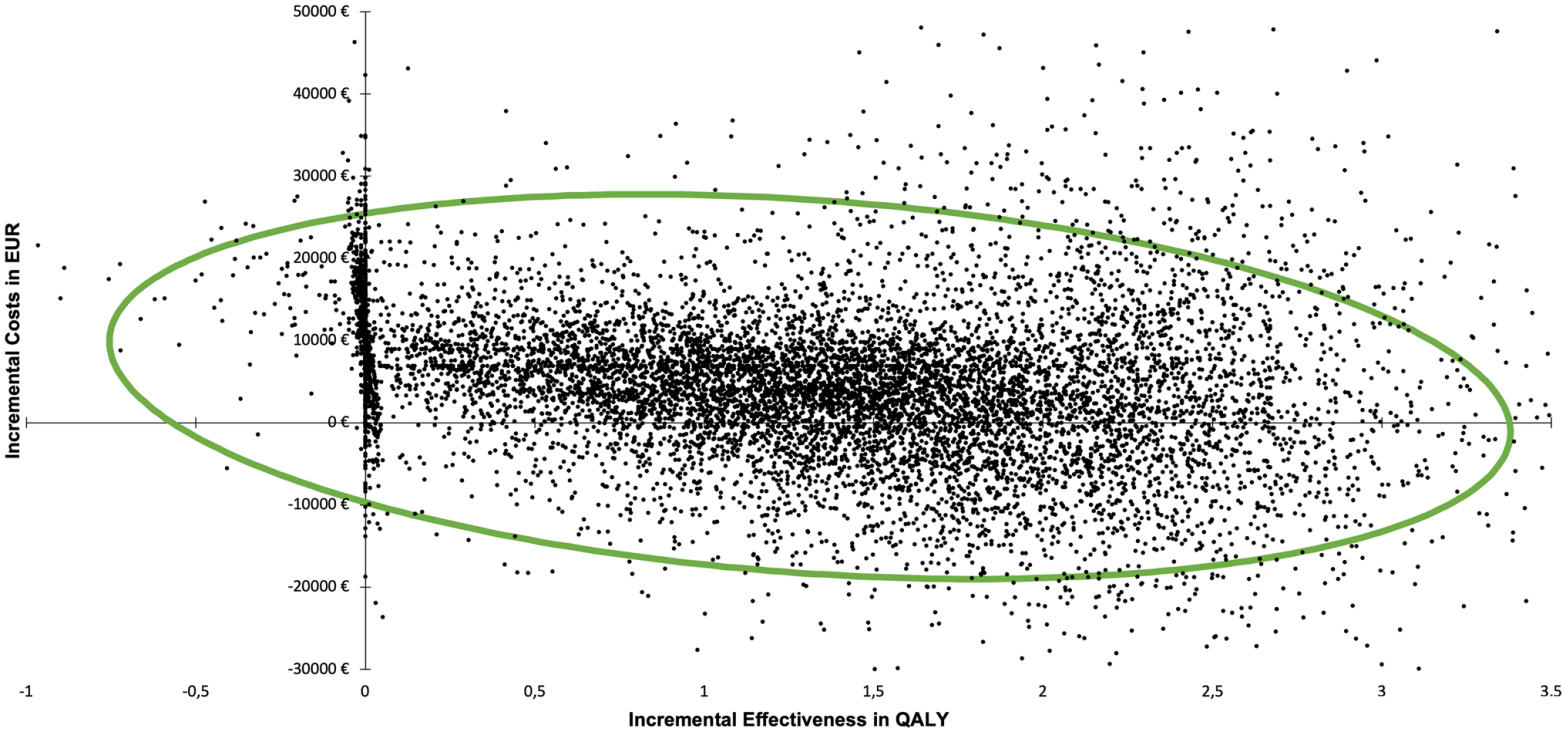
Incremental cost-effectiveness of M-ACI vs. non-M-ACI scenarios

Average QALYs in the 36 years of follow-up were 22.53 in the M-ACI scenario and 21.21 in the scenario without M-ACI, resulting in an incremental QALY gain of 1.32 in favor of the M-ACI scenario. Discounted costs per QALY gained were therefore 3376 € (4455 € cost difference divided by 1.32 difference in QALYs). Assuming a conservative cost-effectiveness threshold of 20,000 € per QALY gained, M-ACI would be deemed cost-effective in 81.9% of all cases; it was found to be dominant, i.e., featuring lower total costs and higher QALYs, in 27.1% of cases (2710 simulated patients) (Fig. 3).

**Fig. 4:**
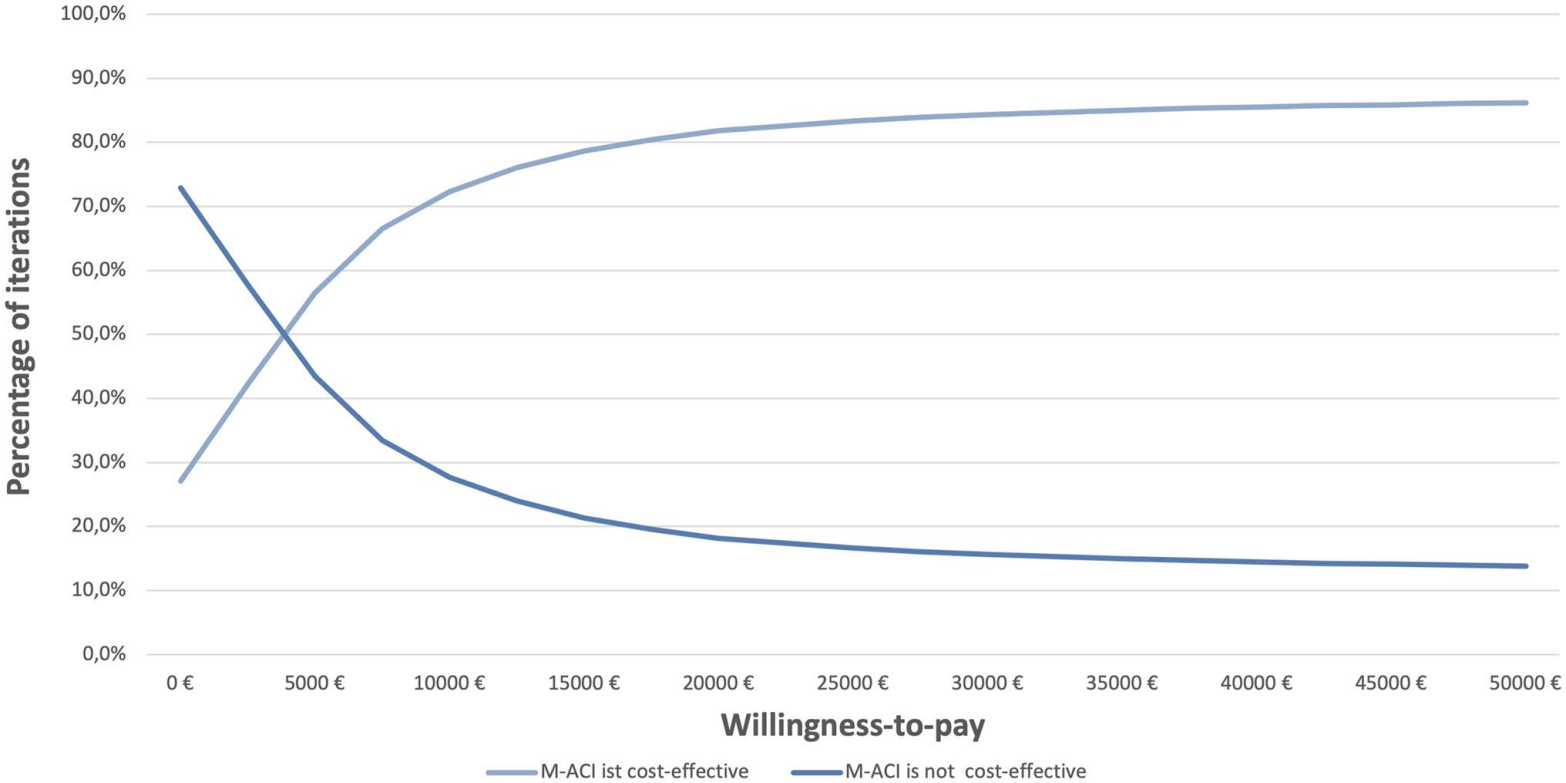
Cost-effectiveness acceptability curve (CEAC) for treatment options. CEAC represent the probability for each scenario of being the most cost-effective option for different WTP thresholds. WTP is the maximum amount the healthcare system would be willing to pay

M-ACI scenario is cost-effective in more than 50% of cases if willingness to pay per QALY for the German healthcare system exceeds 5000 € per QALY. If willingness to pay exceeds 20,000 € per QALY gained, M-ACI is a cost-effective treatment alternative in more than 80% of cases (Fig. 4).

#### Sensitivity of results

**Fig. 5:**
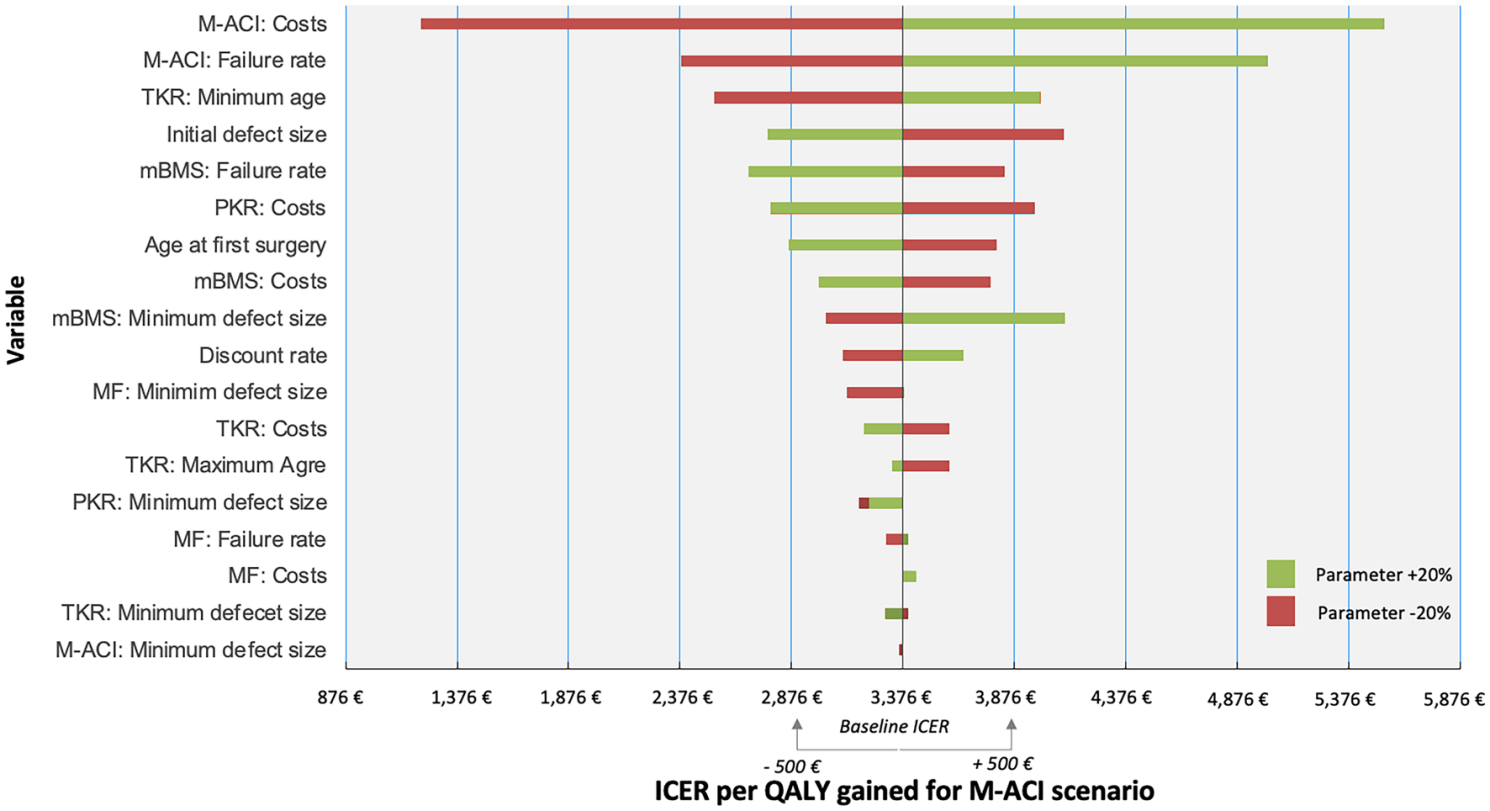
Tornado plot showing the effect of the uncertainty surrounding the input parameters on the ICER per QALY gained for M-ACI scenario. Parameters were varied ± 20% from baseline scenario, the baseline values for the parameters used for this analysis can be found in Table 1

The univariate sensitivity analysis shows robust results for the ICER per QALY (Fig. 5). M-ACI failure rates and costs for M-ACI procedure had the largest impact on the cost-effectiveness of this technology’s application in the treatment process of cartilage defects in the knee. Within the range of ± 20% change, no parameter change led to an ICER per QALY gained of more than 6000 €. The results were also robust for a change in discount rate to 4.5% per year with an ICER of 5348 € (Supplemental material, Table S1) and microfracture as a treatment option for larger defects with an ICER of 4872 € (Supplemental material, Table S2) per QALY gained.

## Discussion

In this modeling study, using patient-individual data on 10,000 M-ACI patients and a discrete event simulation approach, it was shown that 21% TKR can be avoided by using M-ACI in the overall population instead of individual other forms of knee cartilage repairs.

This drives quality of life of patients, who gain 1.32 QALY when operated with M-ACI as compared to a situation without the availability of M-ACI [36]. The increase in total lifetime costs is moderate, namely € 3741 (undiscounted) viz. € 4455 (discounted). At an ICER of €3,376 per QALY gained, the increase in lifetime quality of life would seem to justify the extra spending, as even a very strict willingness-to-pay (WTP) threshold, such as €20,000/QALY gained (largely exhausted by many other means of treatment reimbursed under the German SHI scheme) would be undercut in over 80% of all cases. Already low willingness-to-pay thresholds as 10,000 € lead to M-ACI being cost-effective in more than 70% of cases. Furthermore, M-ACI showed as cost-effective in 50% of cases even if willingness to pay per QALY gained would be as low as 5000 €. This high cost-effectiveness also showed as robust to one-way sensitivity analyses: Altering input parameters by plus/minus 20% did in no case results in ICERs exceeding 6000 € per QALY gained. Our model was most sensitive to altering the failure rate and costs of M-ACI. If the failure rate is 20% higher than in our base case, where we assumed 7% failure after 2 years, ICER per QALY gained would rise from 3376 to 5015 €. Also, if costs of M-ACI would be 20% higher (i.e. 13,200 € instead of 11,000 €), M-ACI would still be an cost effective treatment with an ICER per QALY gained of 5540 €. No parameter change, including discount rates for costs and effects in this lifelong model and changes in indication for microfracture, did alter the results in a way that could question the cost-effectiveness of M-ACI.

Our results are well in line with the international literature, especially from Markov models in other European countries: In a NICE assessment [4], net QALY gains for M-ACI were between 0.0070 (18.189 QALY with M-ACI vs. 18.119 QALY without) and 2.410 (18.189 QALY vs. 15.779 QALY), with costs per incremental QALY of 4360 GBP (approx. 5100 €).

Our study confirms previous estimates, based on German health claims data, namely an ICER per QALY gained over a lifetime lower than the GBP 4360 [14]. Our study also confirms recent works that found that M-ACI is associated with significantly less re-operations, especially compared to microfracture, in the German healthcare setting [37].

Our analysis was based on a number of assumptions and has some limitations. First the quantification of the intervention effects was taken from different studies and not from head-to-head analyses. It cannot be excluded that patient characteristics differed between the different trials used to inform the model.

Another limitation was that the durability of the interventions was modeled from historical data, which may be underestimating the current survivorship of interventions given the technological advancement over decades. This issue has potential impact on cost-effectiveness of the different interventions, since an improved durability of would reduce the number of revisions. Furthermore, the model focussed on the treatment of the initial defect that might re-occur, but did not model new emerging defects explicitly, although new defects are included in the model implicitly as some re-interventions labeled as “revisions” in our model might be attributed to newly emerged defects.

Taken together, M-ACI is projected to be a highly cost‐effective treatment for chondral defects of the knee in the German healthcare setting. Further research is required to explore clinical long-term effects of cartilage repair and reduce uncertainty on long-term quality of life.

## Supplementary Information

Below is the link to the electronic supplementary material.

Supplementary file1 (DOCX 1084 KB)

## Data Availability

No new data were created or analyzed in this study.
