# Supplementary material for: Long-term cost-effectiveness of matrix-associated chondrocyte implantation in the German health care system: a discrete event simulation
Source: Arch Orthop Trauma Surg. 2022 Jan 22;143(3):1417–27. doi: 10.1007/s00402-021-04318-9 (PMC9957880; doi:10.1007/s00402-021-04318-9)
Supplement: Supplementary file 1 — Supplementary file1 (DOCX 1084 KB) [file 402_2021_4318_MOESM1_ESM.docx]

# Long-term cost-effectiveness of matrix-associated chondrocyte implantation in the German health care system: a discrete event simulation

# Supplementary material

**Figure S1. Outline of the discrete event simulation. TKR: Total knee replacement, M-ACI: Matrix-induced autologous chondrocyte implantation, mBMS: matrix-associated bone marrow stimulation, cm: centimeter**


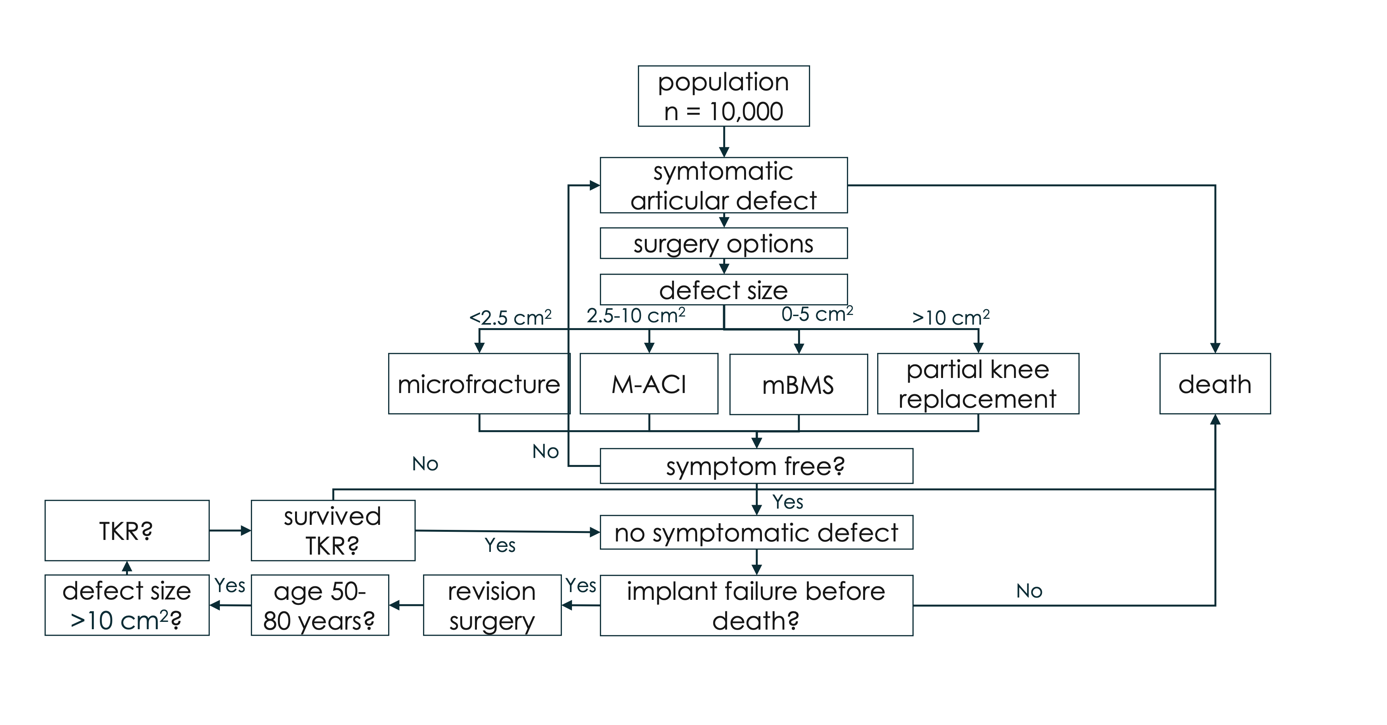


**Figure S2. Legend: Discounted cost differences between the different scenarios in the follow-up period**


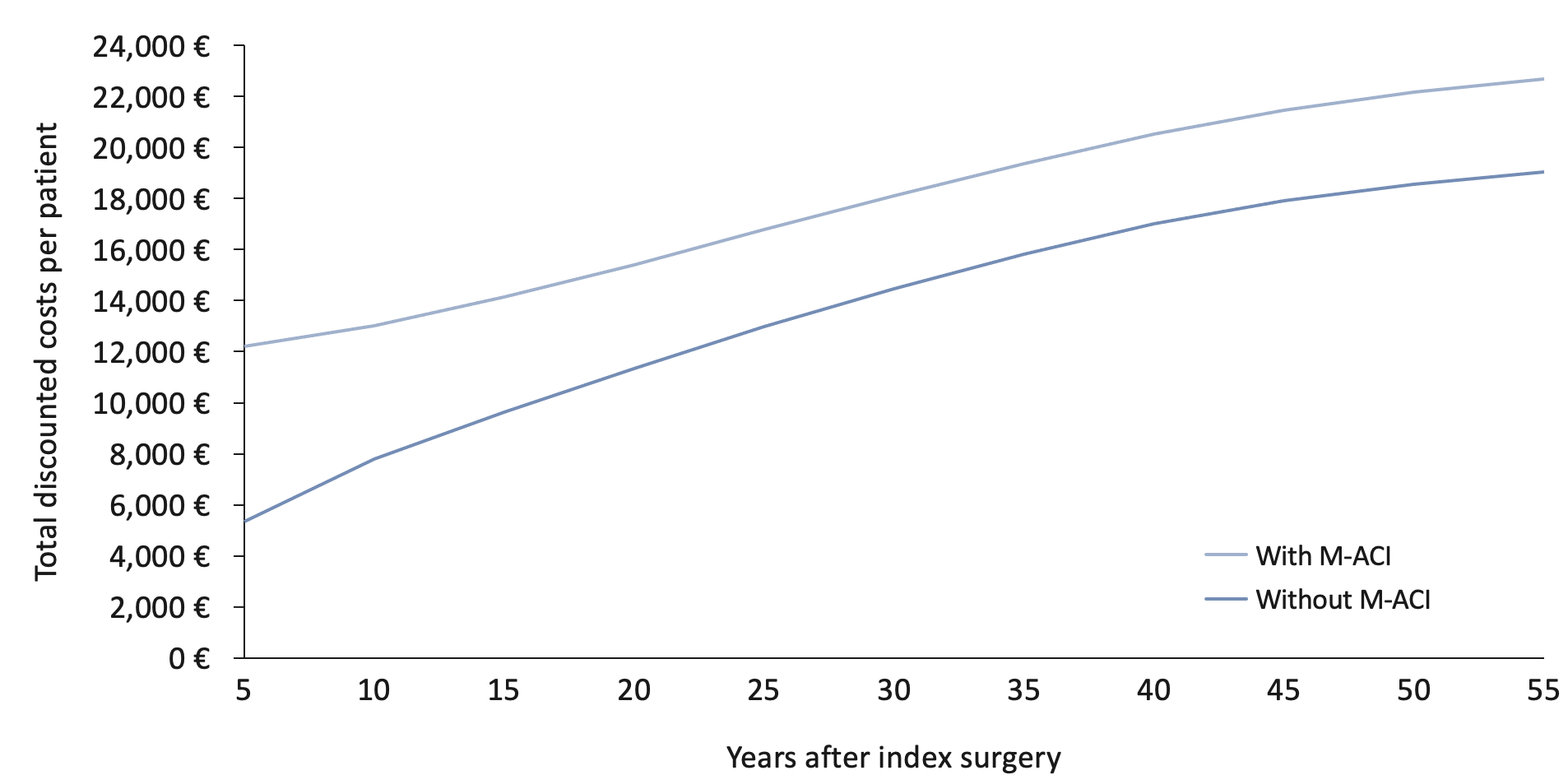


**Figure S3. Weibull estimation for long-term M-ACI failure**

**Figure S4. Weibull estimation for short-term M-ACI failure**

**Figure S5. Weibull estimation for microfracture failure**

**Figure S6. Weibull estimation for mBMS failure**


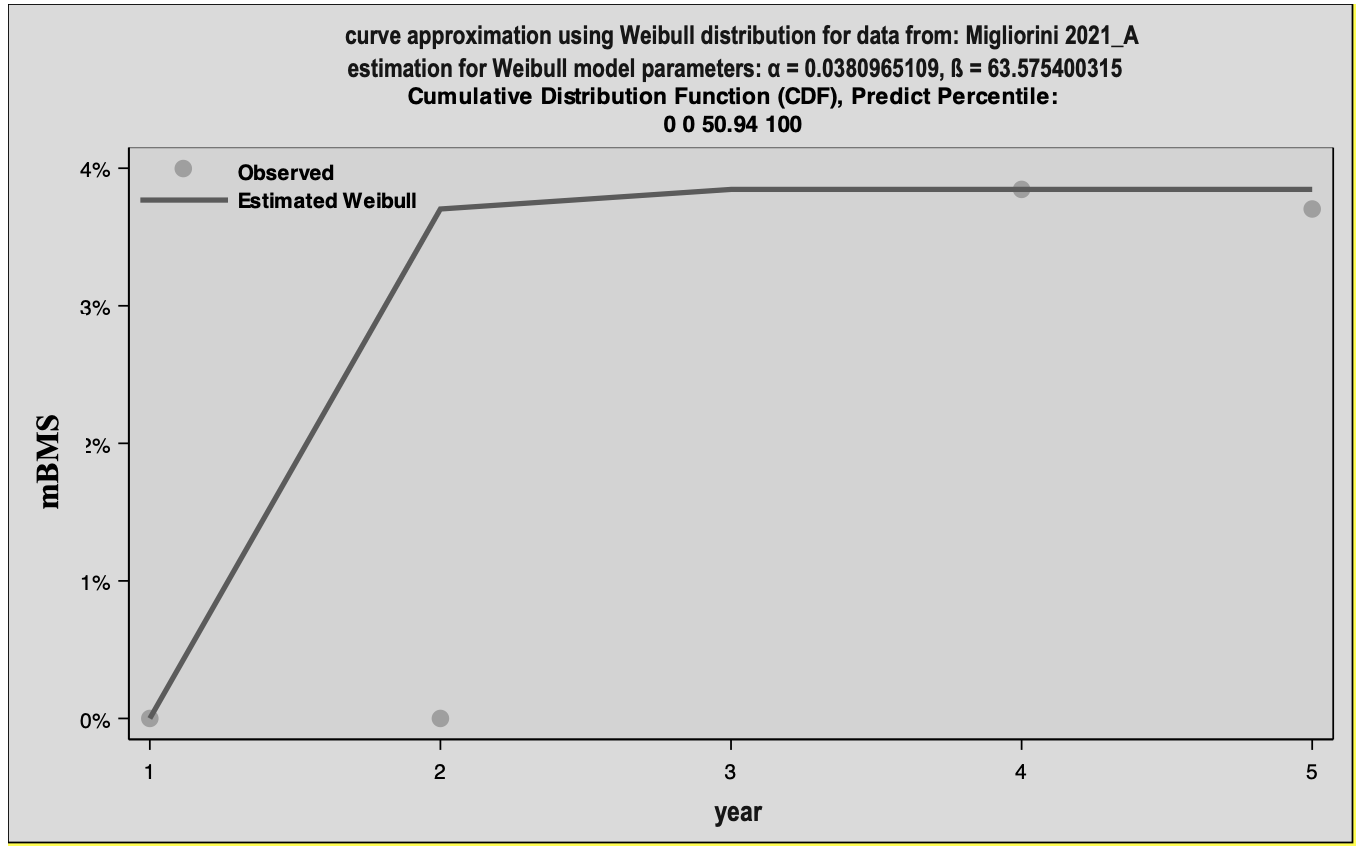


**Figure S7. Weibull estimation for short-term TKR failure**

**Figure S5. Weibull estimation for short-term TKR failure**

**Figure S8. Weibull estimation for long-term TKR failure**

**Table S1. Sensitivity analysis: Discount rate 4.5% for costs and effects**

| *Scenario* |  |  |
| --- | --- | --- |
|  | Original (Costs / Effects 2%) | Costs/Effects 4.5% |
| *With M-ACI* |  | |
| Number of kneeTEP | 5.50% | 5.50% |
| QALY | 22.53 | 14.74 |
| Costs | 18590 | 15,721 € |
| *Without M-ACI* |  | |
| Number of kneeTEP | 26% | 26% |
| QALY | 21.21 | 13.77 |
| Costs | 14,134 € | 10,533 € |
| *Incremental M-ACI vs. Non M-ACI* |  | |
| Incremental QALY | 1.32 | 0.97 |
| Incremental costs | 4,456 € | 5,188 € |
| ICER per QALY | 3,376 € | 5,348 € |

**Table S2. Sensitivity analysis: Microfracture is possible for all defects until 5cm^2^**

| *Scenario* |  |  |
| --- | --- | --- |
|  | Original (Microfracture for defect sizes ≤ 2 cm^2^) | Microfracture for defect sizes ≤5 cm^2^ |
| *With M-ACI* |  | |
| Number of kneeTEP | 5,50% | 6,10% |
| QALY | 22.53 | 22.46 |
| Costs | 18,590 € | 16.578 € |
| *Without M-ACI* |  | |
| Number of kneeTEP | 26% | 29% |
| QALY | 21.21 | 21.24 |
| Costs | 14,134 € | 10,634 € |
| *Incremental M-ACI vs. Non-M-ACI* |  | |
| Incremental QALY | 1.32 | 1.22 |
| Incremental costs | 4,456 € | 5,944 |
| ICER per QALY | 3,376 € | 4,872 € |
